# Uniqueness of Iris Pattern Based on the Auto-Regressive Model

**DOI:** 10.3390/s24092797

**Published:** 2024-04-27

**Authors:** Natalia A. Schmid, Matthew C. Valenti, Katelyn M. Hampel, Jinyu Zuo, Priyanka Das, Stephanie Schuckers, Joseph Skufca

**Affiliations:** 1 Lane Department of Computer Science and Electrical Engineering, West Virginia University, Morgantown, WV 26506, USA; matthew.valenti@mail.wvu.edu (M.C.V.);; 2 HID Global, Austin, TX 78753, USA; 3 Department of Electrical and Computer Engineering, Clarkson University, Potsdam, NY 13699, USA; sschucke@clarkson.edu; 4 Department of Mathematics, Clarkson University, Potsdam, NY 13699, USA; jskufca@clarkson.edu

**Keywords:** sphere packing bound, maximum population, IrisCode, auto-regressive model, Burg’s spectrum estimation, binary detection problem

## Abstract

In this paper, we evaluate the uniqueness of a hypothetical iris recognition system that relies upon a nonlinear mapping of iris data into a space of Gaussian codewords with independent components. Given the new data representation, we develop and apply a sphere packing bound for Gaussian codewords and a bound similar to Daugman’s to characterize the maximum iris population as a function of the relative entropy between Gaussian codewords of distinct iris classes. As a potential theoretical approach leading toward the realization of the hypothetical mapping, we work with the auto-regressive model fitted into iris data, after some data manipulation and preprocessing. The distance between a pair of codewords is measured in terms of the relative entropy (log-likelihood ratio statistic is an alternative) between distributions of codewords, which is also interpreted as a measure of iris quality. The new approach to iris uniqueness is illustrated using two toy examples involving two small datasets of iris images. For both datasets, the maximum sustainable population is presented as a function of image quality expressed in terms of relative entropy. Although the auto-regressive model may not be the best model for iris data, it lays the theoretical framework for the development of a high-performance iris recognition system utilizing a nonlinear mapping from the space of iris data to the space of Gaussian codewords with independent components.

## 1. Introduction

The uniqueness of iris biometrics and methods to evaluate it have been central themes of multiple publications [1,2,3,4]. Most often, iris uniqueness is analyzed assuming a convenient probability model for encoded iris data followed by the development of a bound on the probability of iris recognition error displayed as a function of distortion in the data. For instance, the data projected into PCA and ICA spaces are often modeled as Gaussian distributed with independent components [2,4]. Different techniques have been applied to analyze the uniqueness of binary codes. In [1], the individuality of the iris (a concept relevant to uniqueness) is analyzed by counting the probability of bit flips, concluding how the number of flipped bits affects two conditional probabilities of error, false accept and false reject rates. In [5], iris recognition error rates are evaluated numerically relying on large datasets available for the performance analysis. Daugman’s approach to iris uniqueness is perhaps the most accepted methodology proposed thus far. Daugman’s earlier publications [6,7,8] define uniqueness as the ability of an iris recognition system to enroll more and more classes with the probability of collision between new and enrolled classes near zero. Over the years, his view on iris uniqueness has changed. His latest definition of iris uniqueness (see [9,10] for details) is based on the analysis of a closed iris biometric system. Given an iris database of *M* enrolled classes, uniqueness is quantified by evaluating the chance that iris images from any two randomly selected classes match. Both definitions of uniqueness were applied to assess the uniqueness of IrisCode [6,10,11].

Daugman’s analysis applies to IrisCodes where iris templates are composed of bits. In this case, the distance between iris templates is conveniently measured by calculating the Hamming distance. It is shown by Daugman that a binomial probability mass function (pmf) with approximately 249 degrees of freedom can be fitted into the histogram of imposter Hamming distances, which leads to a new interpretation of the problem of finding the maximum population size of IrisCode. One can think of the existence of a hypothetical mapping from the space of IrisCode to a new space of binary codewords. In the new space, each codeword comprises 249 independent bits, and the Hamming distance between any two codewords is equal to 249. Given that each class is represented by a unique binary codeword with the properties above, an elegant performance analysis invoking limits of error correction codes and an asymptotic case of pairwise binary detection problems easily follows.

As a generalization of Daugman’s approach, in our previous work [12], we use rate-distortion theory (limits of error-correction codes) to establish bounds on the maximum possible population of iris classes that Daugman’s IrisCode can support and display each bound as a function of the Hamming distance between codewords of distinct iris classes. This generalization became possible due to the abovementioned hypothetical mapping of IrisCode templates to a new space of binary codewords, with independent bits, having the property that the distance between any two codewords in the new space is equal to the Hamming distance between two IrisCode templates of two different iris classes. Daugman’s analysis, as well as our previous work, apply exclusively to IrisCodes, that is, iris templates composed of bits, leaving any other type of template out of consideration. Are there other interpretable models that can be analyzed following a well-established rate-distortion theoretical framework? Our goal here is to suggest a basic theoretical framework for the manipulation of non-Gaussian iris data into Gaussian to analyze the uniqueness of the iris biometric modality based on the suggested model and well-established information-theoretical results for Gaussian codewords [13].

As a potential theoretical approach leading toward the realization of the hypothetical mapping, we work with the auto-regressive (AR) model fitted into iris data, after some data manipulation and preprocessing. The AR model is designed with sufficient flexibility to encapsulate a broad spectrum of iris patterns, offering several advantages that allow for a theoretical framework suitable for high-performance iris recognition systems. The AR model-based theoretical framework relies upon a hypothetical mapping of iris data into a space of Gaussian codewords with independent components. The distance between a pair of codewords is measured in terms of the relative entropy (log-likelihood ratio statistic is an alternative) between distributions of codewords, which is also interpreted as a measure of iris quality. We develop and apply a sphere packing bound for Gaussian codewords and a bound similar to Daugman’s to characterize the maximum iris population as a function of the relative entropy (log-likelihood ratio statistic) between Gaussian codewords of distinct iris classes. The new approach to iris uniqueness is illustrated using two basic examples involving two small datasets of iris images. For both datasets, the maximum sustainable population is presented as a function of image quality expressed in terms of relative entropy.

To provide more details, our analysis of iris uniqueness assumes fitting the AR model into iris data. When driven by a white Gaussian noise process, the AR model generates a stationary Gaussian random process unique for each iris class. Given a random description of each iris class, the problem of iris recognition is restated as an M-ary detection problem, which is further simplified by replacing it with a union of pairwise binary detection problems. The log-likelihood ratio test statistic in asymptotic form is implemented for each pair of classes, which, when averaged over a large number of images per class, can be substituted with an estimate of the relative entropy between a pair of jointly Gaussian probability density functions each with zero mean and estimated power spectral density. It is further justified that the histogram of the pairwise log-likelihood ratios can be fitted with a chi-square curve. Its degrees of freedom and the scaling factor are determined by minimizing the least square distance (it performs similarly to the chi-square test) over a broad range of the two parameters. Similar to how Daugman interprets 249 degrees of freedom of the fitted binomial curve as the length of hypothetical binary codewords with independent bits, we interpret the degrees of freedom of the fitted chi-square curve as the length of hypothetical Gaussian codewords and the scaling parameter of the fitted chi-square curve as the variance of each codeword entry. The entries of the codewords are independent and identically distributed.

Given the fitted chi-square model, two different approaches to quantify the uniqueness of iris biometrics are presented: (1) A sphere packing argument for the Gaussian source is applied first. The log-likelihood ratio statistic and the estimate of the relative entropy between two iris classes are N-Erlang distributed. This distribution can be alternatively thought of as being due to a sum of 2N squared independent identically distributed real-valued Gaussian random variables. We can fit a chi-square distribution with 2N degrees of freedom, then apply a sphere packing argument to find the dependence of the maximum population and the distortion in the data. (2) It is followed by a Daugman-like bound where the false match rate (FMR) [7] is replaced by the estimated relative entropy or by the log-likelihood statistic averaged over multiple images of each of the two classes.

By integrating the AR model with the union bound-based analysis, the large-scale performance of iris recognition systems may be inferred from limited data. The AR model captures the variability in iris features in a manner that is sufficient when paired with the union bound analysis, which estimates the upper limits of error rates. Together, the approach enables accurate performance predictions without the need for extremely large datasets. Hence, this methodology not only circumvents the practical challenges of extensive data collection but also provides insights into system scalability and efficiency. By leveraging these analytical tools in tandem, we present a novel, efficient framework for predicting the robustness and reliability of iris recognition systems as they scale, marking a significant contribution to biometric system evaluation.

The rest of the paper is organized as follows: Section 2 presents the assumptions, model, and theory. Section 3 presents a toy example (a much simplified argument) of how we can arrive at the theorized model in practice, and presents the illustrative results of performance analysis, including a sphere packing bound and a Daugman-like bound in application to two small iris datasets. Section 4 concludes the paper.

## 2. Theory, Model, and Analysis

Assume that each iris class can be described by a piece-wise stationary Gaussian random process and that the enrollment data of each iris class are a finite sample realization of a class random process. The auto-regressive (AR) process is an example of a stationary random process that can be used to model iris data. With a Gaussian random process for each iris class, we can state the problem of iris recognition as an M-ary detection problem and then apply a variety of analytical tools to analyze the performance of a large iris biometric population.

### 2.1. AR Model for Vectorized Iris Images

Let *M* be the number of enrolled iris classes and *N* be the number of images per class. When analyzing performance, we assume that the same number of images is available per iris class to avoid any unwanted performance bias. We further assume that iris images are conveniently vectorized. Let $X_{1}^{n}\left(m\right),\ldots ,X_{N}^{n}\left(m\right)$ be *N* vectorized iris images of iris class m,m=1,…,M, with superscript *n* indicating the length of each vector. Note that in our analysis, we treat all vectors as column vectors.

To ensure a workable model that can be used to analyze the performance of iris biometrics, we turn to an auto-regressive (AR) model [14] for vectorized iris data. As a model, AR has two outstanding properties. (1) It is driven by white Gaussian noise passed as an input to a linear shift-invariant filter. Furthermore, (2) the model captures dependencies among entries in $X_{i}^{n}\left(m\right);$ that is, the entries in a vector $X^{n}$ are related through the following equation:

(1) $$X_{t}=\sum_{i=1}^{p}\alpha_{i}X_{t-i}+\eta_{t},$$

where $\alpha_{i}$ is the parameter of the model, $\eta_{t}$ is a sample of the white Gaussian noise process with mean zero and variance $\sigma_{\eta }^{2},$ and *p* is the parameter that determines the order of the model.

If the AR model does not provide a reasonable fit to the vectorized data, several variants of AR, such as applying it to log or exponentially transformed data or to high-order difference data (AR(Integrated)MA) [14], may lead to a better fit.

Noting that we are dealing with a linear difference equation and, thus, with a description of a linear system, the frequency response of the AR model in (1) is easy to derive

(2) $$H\left(f\right)=\frac{1}{1+\sum_{k=1}^{p}\alpha_{k}\exp\left(-j2\pi fk\right)},$$

where we use *f* to denote frequency.

Since the AR model describes the stationary behavior of data (usually time or spatial series), by knowing the transfer function of the model and the power spectrum of the driving process (which in our case is a white Gaussian noise process with zero mean and variance $\sigma_{\eta }^{2}$), we can write an equation of the power spectral density (PSD) of the data $X_{t}$

(3) $$S_{X}\left(f\right)=\sigma_{\eta }^{2}{\left|H\left(f\right)\right|}^{2},$$

where $S_{X}\left(f\right)$ is the notation for the PSD on the output of the linear filter.

Given an AR model, each iris vector $X_{i}^{n}\left(m\right)$ is a realization of the random process described by (1). Since the random process is driven by a Gaussian noise process and the model is linear, the process in (1) is also Gaussian. Thus,

(4) $$X_{i}^{n}\left(m\right)∼N\left(\mu \left(m\right),K\left(m\right)\right),$$

where μ(m) is the mean (in our analysis, we adjust it to be 0) and K(m) is the covariance matrix of the entries of the *i*-th vectorized iris image of the *m*-th class. Each iris class is fitted with a unique AR model.

### 2.2. Classical Approach to the Estimation of Maximum Population

Given probability models for the data of each class and the class dependencies, an optimal approach to the analysis of iris uniqueness is to state the problem of matching a query iris image $Y^{n}$ to one of *M* iris classes as an *M*-ary detection problem [15]. A direct performance analysis for this problem requires forming an (M−1) dimensional vector of likelihood ratios and evaluating their joint probability density under the assumption that the query data belong to one of *M* distinct iris classes. Mathematically, performance analysis for this problem becomes quickly intractable, since the expression for the joint probability density function of the vector of likelihood ratios is not straightforward to develop. Furthermore, it is hard to implement in practice. Seeking for an alternative solution, one may turn to an analysis of M(M−1)/2 binary detection problems, an approach that is often used in practice. By applying the union bound [16], the probability of error in an M-ary problem can be upper bounded by a sum of binary error probabilities.

Denote by P(error) the average probability of error in an *M*-ary detection problem and by $P(error|H_{m})$ the conditional probability of error. Given that data are generated by Class m,m=1,…,M, we refer to it as hypothesis $H_{m}.$ Assuming equal prior probability for each class m, the average probability of error is given as

(5) $$P\left(error\right)=\frac{1}{M}\sum_{m=1}^{M}P\left(error|H_{m}\right).$$

After expanding $P(error|H_{m})$ as $P({⋃}_{k=1,\;k\neq m}^{M}H_{k}|H_{m})$ and applying the union bound, the equation above yields

(6) $$P\left(error\right)\leq \frac{1}{M}\sum_{m=1}^{M}\sum_{k=1,\;k\neq m}^{M}P\left(H_{k}|H_{m}\right),$$

where $P(H_{k}|H_{m})$ is the error in a binary detection problem for the pair of classes *k* and m.

The bound (6) establishes a link between the total probability of recognition error and the number of iris classes *M*, and, thus, presents a basis for the analysis of the maximum population of iris biometrics. Despite being much simplified compared to the original *M*-ary detection problem, the bound does not yield a general explicit relationship between P(error) and *M* and becomes hard to evaluate in practice due to the complex nature of practical data.

To take our analysis of the maximum iris population further, in the following subsections, we will first develop an expression for the log-likelihood ratio statistic and analyze its probability distribution. Then, we will return to the bound on P(error) and suggest two alternative approaches that yield an explicit relationship not only between P(error) and M, but also involving the quality of iris data (see [11] for the definitions and standards on iris quality for iris biometrics).

### 2.3. Log-Likelihood Ratio

Given an iris dataset composed of *M* iris classes, with the data of each class being vectorized and then fitted with an AR description, as outlined in Section 2.1, the origin of a query vector $Y^{n}$ can be tested using classical detection theory approaches. Since we have a probability model for data of each class, however, the parameters of the models are estimated from data; we appeal to the generalized likelihood ratio test (GLRT) [15] to find which of *M* classes is the origin of vector $Y^{n}.$ While our peers may find this approach outdated (too classical compared to modern deep learning-based approaches), unlike deep learning approaches, this model guarantees an insightful performance analysis, which is a powerful justification within the scope of this work.

Given M(M−1)/2 pairwise binary detection problems to solve, we form a log-likelihood statistic for every pair. For testing the hypothesis “class *m* is the true class” versus “class *k* is the true class”, it is given as

(7) $$\Lambda \left(m,k\right)=\frac{1}{N}\sum_{j=1}^{N}\ln\frac{f(Y_{j}^{n}|H_{m})}{f(Y_{j}^{n}|H_{k})},$$

where $f(Y_{j}^{n}|H_{m})$ is the conditional pdf of the *j*-th copy of vectorized iris data $Y^{n},$ conditioned on class m. After involving the model in (4), the log-likelihood statistic becomes

(8) $$\Lambda \left(m,k\right)=-\frac{1}{2N}\sum_{j=1}^{N}Y_{j}^{nT}\left(K^{-1}\left(m\right)-K^{-1}\left(k\right)\right)Y_{j}^{n}-\frac{1}{2}\ln\det\left(K\left(m\right)K^{-1}\left(k\right)\right).$$

The test statistic Λ(m,k) is then compared to a threshold to conclude which class “generated” the vector $Y^{n}.$ We tentatively set the value of the threshold to zero, since no prior information about the frequency of use of any two classes is available to us, and thus the binary test to perform is given as

(9) $$\Lambda \left(m,k\right)\underset{H_{m}}{\overset{H_{k}}{≶}}0.$$

Alternatively, we can vary the value of the threshold on the right-hand side of the inequality and analyze $P(H_{k}|H_{m})$ as a function of the threshold.

### 2.4. Asymptotic Case of Log-Likelihood Ratio

When the number of entries in a vectorized iris image is large, that is, *n* is large, (8) can be replaced by an asymptotic expression involving the power spectral density of the AR model. It can be easily demonstrated that Λ(m,k) in the asymptotic case can be written as

(10) $$\Lambda \left(m,k\right)=-\sum_{i=0}^{n-1}\left\{\left(\frac{1}{S_{m}\left(f_{i}\right)}-\frac{1}{S_{k}\left(f_{i}\right)}\right)\sum_{j=1}^{N}\frac{|y_{j}\left(f_{i}\right){|}^{2}}{N}+\ln\left(\frac{S_{m}\left(f_{i}\right)}{S_{k}\left(f_{i}\right)}\right)\right\}=-\sum_{i=0}^{n-1}\lambda \left(f_{i}\right),$$

where $y^{n}$ is the Fourier transform of $Y^{n},$ $S_{m}\left(f_{i}\right)$ is the *i*-th sample of the power spectral density of the *m*-th class (for an insightful explanation of the result, see p. 36 of Kay [17]), and $\lambda (f_{i})$ is the *i*-th component of the log-likelihood ratio statistic.

### 2.5. Analysis of Error Probability, Continued

Given a binary detection problem involving two classes, Classm and Classk, an error will occur in two cases: Case 1: $Y^{n}$ originated from Classm, but Λ(m,k)<0; and Case 2: $Y^{n}$ originated from Classk, but Λ(m,k)>0. The first case describes $P(H_{k}|H_{m}),$ while the second case describes $P(H_{m}|H_{k}).$ Both conditional probabilities of error can be expressed in terms of the conditional probability density function of Λ(m,k), assuming one or the other class is the true class.

Consider $P\left(H_{k}|H_{m}\right)=P\left(\Lambda \left(m,k\right)<0|H_{m}\right).$ In (10), random vector $y^{n}$ is complex Gaussian under either hypothesis, since $y^{n}$ is a linear transformation of a Gaussian vector. To find the conditional probability of error $P(H_{k}|H_{m}),$ we need a closed form expression for the conditional probability density function (pdf) of Λ(m,k) under $H_{m}.$

Assuming that $y^{n}$ is from Class *m* implies that $y\left(f_{i}\right)∼\mathrm{CN}\left(0,S_{m}\left(f_{i}\right)\right),$ where CN denotes “complex normal”,

$$y\left(f_{i}\right){\left(\frac{1}{S_{m}\left(f_{i}\right)}-\frac{1}{S_{k}\left(f_{i}\right)}\right)}^{1/2}∼\mathrm{CN}\left(0,S_{m}\left(f_{i}\right)\left(\frac{1}{S_{m}\left(f_{i}\right)}-\frac{1}{S_{k}\left(f_{i}\right)}\right)\right)$$

and

$$\lambda \left(f_{i}\right)=\left(\frac{1}{S_{m}\left(f_{i}\right)}-\frac{1}{S_{k}\left(f_{i}\right)}\right)\sum_{j=1}^{N}\frac{|y_{j}\left(f_{i}\right){|}^{2}}{N}+\ln\left(\frac{S_{m}\left(f_{i}\right)}{S_{k}\left(f_{i}\right)}\right)$$

is a *N*-Erlang random variable with the pdf

(11) $$f_{\lambda (f_{i})}\left(x\right)=\frac{N^{N}}{{\left(\sigma_{i}^{2}\right)}^{N}}\frac{{\left(x-a_{i}\right)}^{N-1}}{(N-1)!}\exp\left\{-\frac{N(x-a_{i})}{\sigma_{i}^{2}}\right\},\;\;x>a_{i}$$

where $a_{i}=\ln\left(S_{m}\left(f_{i}\right)/S_{k}\left(f_{i}\right)\right)$ and $\sigma_{i}^{2}=1-S_{m}\left(f_{i}\right)/S_{k}\left(f_{i}\right).$

The entries $\lambda (f_{i})$ in the test statistic Λ(m,k) are independent, but not identically distributed. Therefore, a closed-form expression for the conditional pdf of Λ(m,k), assuming that the data are generated by Classm, is not straightforward to find. At this point, we can take our analysis further by involving the Chernoff bound [15] on $P(H_{k}|H_{m}).$ Instead, equipped with the form of the pdf for $\lambda (f_{i}),$ the well-developed theory of error correction codes [13,18], and a deep insight into Daugman’s analysis of IrisCode [9,10], we reverse the direction of our analysis. In the following two subsections, we analyze the uniqueness of iris biometrics from the perspective of the sphere packing argument [13] and by developing a Daugman-like bound [9]. Both provide an explicit relationship of P(error) on the number of classes *M* and an average quality of iris data in a considered iris dataset.

### 2.6. Analysis of Iris Uniqueness Using Sphere Packing Argument

As justified in Section 2.5, the log-likelihood ratio test statistic is a sum of weighted exponential random variables. While no method for direct evaluation of its pdf is known, a plot of the relative frequency of the log-likelihood statistic can be approximated by a chi-square pdf formed by adding *K* iid squared complex Gaussian random variables, each with zero mean and variance P. The parameter *K* is the number of degrees of freedom of the fitted chi-square pdf. Since *K* and *P* are unknown, they must be estimated from empirical data.

The fitted chi-square pdf allows us to interpret the problem of finding the maximum iris population as a Gaussian sphere packing result. Suppose an encoding strategy is available to map ideal iris images (iris images with no noise or distortions) of *M* distinct iris classes into unique Gaussian codewords, each of length K. Each codeword is drawn iid from a Gaussian distribution with zero mean and variance P. Suppose further that an iris image of one of the *M* classes (for example, of Class *m*) submitted for authentication or recognition is modeled as a noisy version of the ideal codeword of Class m. The noise is zero mean Gaussian with variance *N* in each of *K* dimensions. Thus, for a given Class *m*, the iris image submitted for authentication is mapped into a point within a *K*-dimensional sphere with radius $\sqrt{KN}$ around the codeword of Class m. Since the Gaussian sphere containing codewords of *M* classes has radius $\sqrt{K(P+N)},$ the maximum number of classes, assuming that the distortion of iris images submitted for authentication is bounded, can be obtained by dividing the volume of a *K*-dimensional sphere containing all codewords by the volume of the small sphere representing noise in the data of a particular iris class. Thus,

(12) $$M\leq {\left(1+\frac{P}{N}\right)}^{K/2}.$$

See [13] for a more insightful description.

### 2.7. A Daugman-like Approach to the Analysis of Iris Uniqueness

Similar to the sphere packing argument presented in the previous section, Daugman-like analysis of iris uniqueness is based on the assumption that the data of iris classes are mapped into a space in which each iris class is presented by an independent Gaussian codeword of length *K* with zero mean and variance P, where *K* and *P* are defined above. This mapping ensures that the asymptotic pairwise log-likelihood ratios (here interpreted as a distance between two codewords) are independent chi-square distributed random variables with *K* degrees of freedom. The asymptotic log-likelihood ratio can also be replaced with an estimate of the relative entropy between the pdfs of two iris classes. This leads to a new interpretation of the distance measure as a means to also measure the quality of iris data. Its introduction allows a rate-distortion interpretation of the problem of finding the maximum iris population that an iris recognition system can sustain, similar to how error-correction bounds in coding theory relate the maximum population of binary code to the minimum Hamming distance between codewords [18]. To be specific, the introduction of such a metric will lead to a new performance bound that relates the size of the iris population covered by the recognition algorithm and the quality of iris biometric data while ensuring a small probability of recognition error.

At this point of our analysis, in addition to the asymptotic log-likelihood ratio statistic, we introduce the relative entropy between the probability density functions of two classes *m* and k. The relative entropy is defined as the expected value of the log-likelihood ratio in (10)

(13) $$d\left(m,k\right)=E\left[\Lambda (m,k)\right]=\sum_{i=0}^{n-1}\left\{\frac{S_{m}\left(f_{i}\right)}{S_{k}\left(f_{i}\right)}-\ln\frac{S_{m}\left(f_{i}\right)}{S_{k}\left(f_{i}\right)}-1\right\},$$

where E is the notation for the expected value operator. Since the power spectral densities of different iris classes are not known to us, they are first estimated from available class data and then plugged in the expression for the relative entropy in place of the true unknown power spectral densities.

With estimated relative entropy as a distance metric, the bound on the maximum population of the enrolled iris population is straightforward to develop. We follow an argument similar to Daugman’s that the imposter distance between a pair of distinct iris classes can be fitted with a chi-square pdf with *K* degrees of freedom. Then, the error to enroll can be mathematically described as

(14) $$P\left(error\;to\;enroll\right)=1-P\left(\overset{M}{\underset{m=1}{⋂}}d\left(m,M+1\right)>\tau \right),$$

where d(m,M+1) is the distance between a previously successfully enrolled class *m* and a new (not yet enrolled) class M+1 and τ is a minimum distance between two codewords for them to represent two distinct classes. Since pairwise distances between iris classes are independent identically distributed chi-square random variables, (14) can be rewritten as

(15) $$P\left(error\;to\;enroll\right)=1-{\left\{1-P(d(m,M+1)\leq \tau )\right\}}^{M}\leq \delta .$$

Inverting the inequality for *M* results in

(16) $$M\leq \frac{\log(1-\delta )}{\log\left\{1-FMR(\tau )\right\}},$$

where P(d(m,M+1)≤τ) is replaced with FMR(τ), an abbreviation for the false match rate as a function of the distance between two codewords τ.

## 3. Illustration of the Methodology

The following section provides a basic illustration of the abovementioned theory on two small subsets of the CASIA-IrisV3 Interval and BATH datasets. As the sphere packing bound (a purely theoretical result) and a methodology to approach it are the main focus of this work, we do not intend to extend their illustration beyond this example to avoid obscuring the theory and methodology.

**Figure 1:**
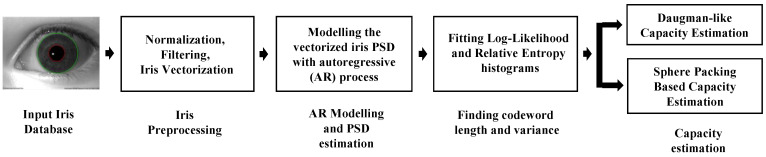
An overall block diagram summarizing the proposed methodology for finding the maximum population of an iris database.

The power spectral densities for each dataset are estimated using the AR modeling described in Section 2.1, along with justification for selecting the correct model order. The estimated power spectral densities for each class are then substituted into the distance metrics in (10) and (13). For both metrics, the histograms are fitted using a chi-square distribution, as outlined in Section 2.6, and the resulting degrees of freedom and variance are substituted into (12) and (16) to find the maximum population based on iris quality and recognition error. Figure 1 shows an overview of the methodology presented and discussed in subsequent sections.

### 3.1. Data

The following illustration is carried out on two small datasets: the Chinese Academy of Sciences’ Institute of Automation (CASIA) CASIA-IrisV3 Interval [19] and the University of Bath (BATH) Iris Image Database [20]. CASIA-IrisV3 Interval contains 2639 near-infrared (NIR) illuminated images, each having a resolution of 320×280 pixels, and a total of 249 subjects. A trial subset of the BATH dataset contains 1000 images, each with resolution 960×1280 pixels, and 25 subjects (with each subject having 20 images for both the left and right eye). These datasets are chosen due to their high-quality iris images that show the rich texture around the pupil.

Some data reduction is performed on each iris dataset to balance the dataset and extract the highest-quality iris images. The CASIA-IrisV3 Interval dataset is reduced by removing images with more than 50% iris occlusion and then excluding classes with fewer than 10 images per class, resulting in only 21 remaining classes with a total of 210 images. After a similar analysis of images in the BATH dataset, 40 iris classes were retained with a total of 800 images. These smaller sets of data are used for the remainder of the paper.

### 3.2. Segmentation and Preprocessing

Iris images are segmented [21], normalized, and Gabor-filtered. Since a majority of the texture of the iris is located close to the pupil, only half of the filtered image is considered and the remainder is discarded. Once preprocessed, the complex-valued image is unwrapped into a single vector (vectorized). We considered multiple methods for vectorization and concluded with Zigzag vectorization [22]. It unwraps the real-valued portion of the image into a one-dimensional vector by applying a diagonal scan from the top left corner of the image to its bottom right corner. The same unwrapping is applied to the imaginary-valued portion of the image, and then the real and imaginary-valued one-dimensional vectors are concatenated into a long data vector (for our data size at 4800 pixels). The applied vectorization may not be the best existing method to vectorize iris images; however, it is suitable enough to illustrate the proposed theory and bounds. For our application, Zigzag helps in reducing the variance in AR coefficients and eliminating induced periodicity in the estimated power spectral density incorporated due to spatial distortion of the iris patterns in horizontal (row-based) or vertical (column-based) vectorization.

### 3.3. Estimation of Power Spectra

As stated in Section 2.1, to ensure a workable model that can be used to analyze the performance of iris biometrics, we turn to an auto-regressive (AR) model for the vectorized iris data. The analysis of maximum population is based on the successful implementation of (10) and (13), which, in turn, rely upon estimates of the power spectral densities obtained from data of iris classes. These estimates are obtained through (i) finding the optimal order for the AR model given our iris data, Section 3.3.1, and (ii) using Burg’s maximum entropy method [23] to find high-quality spectral estimates for each iris class, Section 3.3.2.

#### 3.3.1. Finding Optimal Model Order

Estimating the appropriate model order is essential in the performance of the AR model. Having a large order ensures a better fit into data; however, it also increases the complexity of the implementation and can lead to fitting the AR model to noise rather than to signal. To find the optimal model order of estimated power spectra, we involve the Akaike information criterion (AIC) in conjunction with the AR method in Matlab (Version R2024a).

**Figure 2:**
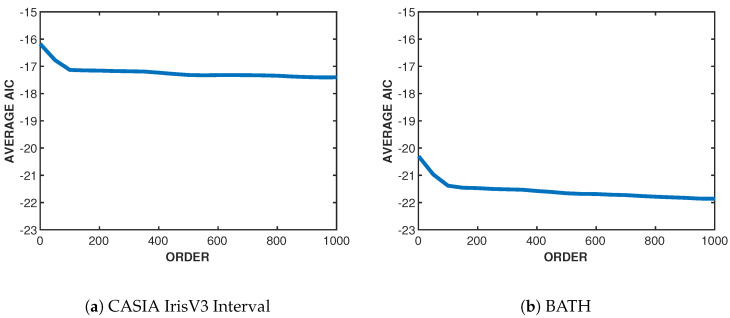
Plots of average AIC values for a single class in (**a**) CASIA-IrisV3 Interval and (**b**) BATH with optimal Gabor filter parameters of $f_{0}=1/9$ and σ=0.5. (AIC scores were found for each iris image separately; then, the average scores were plotted for analysis).

To begin, a subset of iris classes was extracted from each dataset based on varying texture levels, from very fine to rough texture, to see if the structure of the iris affects the model order. Through extensive search, the model order is varied along with two parameters of Gabor filters, the center frequency, $f_{0},$ and the filter bandwidth, σ. The goodness of fit of each estimated model is measured using the value of AIC for each tested model order. This is repeated for each class in the subset on each iris sample until the optimal order, center frequency, and bandwidth are found in which the AIC converges. Figure 2 shows the AIC curves for CASIA-IrisV3 Interval and BATH with the selected order at 100 and Gabor filter parameters of $f_{0}=1/9$ and σ=0.5. Although Figure 2 only shows one iris class for each dataset, we numerically confirmed that each class can be parameterized by the same model order. Choosing a higher order leads to the same performance as an order of 100.

#### 3.3.2. AR Implementation

**Figure 3:**
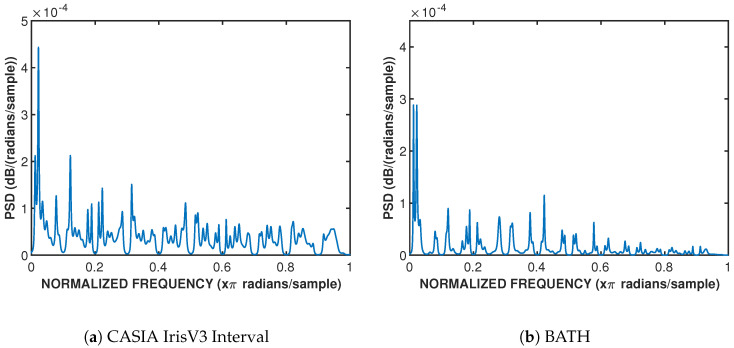
Estimated power spectral densities for both datasets through the use of Matlab’s *pburg* method. Subfigure (**a**) uses CASIA IrisV3 Interval database and subfigure (**b**) uses BATH database.

Burg’s maximum entropy method, which is popular for stationary or piece-wise stationary data, is implemented to find high-quality spectral estimates for each iris class. For both databases, the power spectral densities (PSDs) were estimated for each image in each class through the use of Matlab’s signal processing toolbox function *pburg*, with the found optimal order from Section 3.3.1 as the model order input. The vectorized iris images are found to be low-frequency signals, as shown in Figure 3.

After completion of Burg’s method, a selection of PSDs is averaged to create a high-quality enrollment spectrum for each class, and the remaining spectra are used for the authentication process. The number of samples used for the enrollment spectrum depends on the available amount of iris images per class, with BATH having 20 images available per iris class and CASIA-IrisV3 Interval having a maximum of 10 images. With this in mind, half (50%) of the class’s iris images are used to find the enrollment spectrum, while the remaining percentage is used for authentication. These estimates are used in (10) and (13) to empirically find the maximum population given the sphere packing bound and Daugman-like bound, shown in Section 3.5 and Section 3.6.

### 3.4. Fitting Imposter Distribution

**Figure 4:**
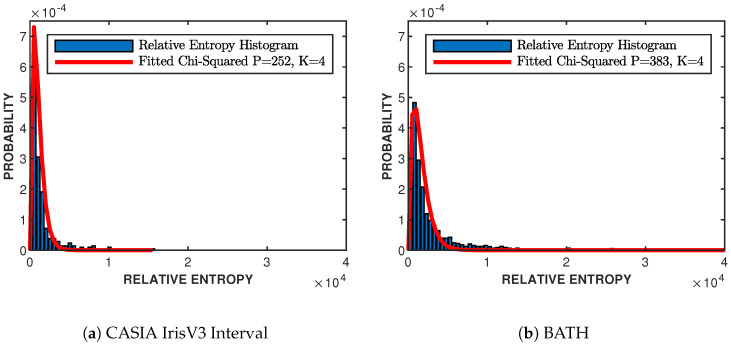
Relative entropy imposter distributions for both datasets with best-of-fit chi-square distributions. Relative entropy is measured in nats. CASIA-IrisV3 Interval having K=4 degrees of freedom and a fitted variance of P=252, shown in (**a**), and BATH having K=4 degrees of freedom and a fitted variance of P=383, shown in (**b**).

**Figure 5:**
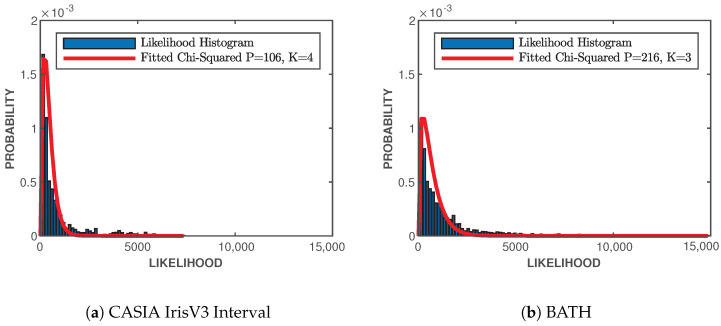
Likelihood imposter distributions for both datasets with best-of-fit chi-square distributions. Likelihood is measured in nats. CASIA-IrisV3 Interval having K=4 degrees of freedom and a fitted variance of P=106, shown in (**a**), and BATH having K=3 degrees of freedom and a fitted variance of P=216, shown in (**b**).

To find the empirical values for *K* and *P* from Section 2.6 and Section 2.7, the imposter distributions of both likelihood values and relative entropies have to be fitted with chi-square distributions. With the use of the estimated PSDs from Section 3.3.2, the pairwise likelihood scores and relative entropies are found for all possible combinations, where $S_{m}$ and $S_{k}$ are from different classes. The best-fit chi-square distribution is found by performing an exhaustive search on the imposter histograms and finding the variance and degrees of freedom that produce the minimum least square error. Figure 4 and Figure 5 show the distributions for both CASIA-IrisV3 Interval and BATH datasets. Both datasets have the best fit with four degrees of freedom, K=4, and different variances of $P_{CASIA}=252$ and $P_{BATH}=383$ for the relative entropy imposter distributions, shown in Figure 4. Figure 5 shows the fitted likelihood distributions with CASIA-IrisV3 Interval having four degrees of freedom, K=4, and a variance of $P_{CASIA}=106$, and BATH having three degrees of freedom, K=3, and a variance of $P_{BATH}=216.$

### 3.5. Sphere Packing Bound

**Figure 6:**
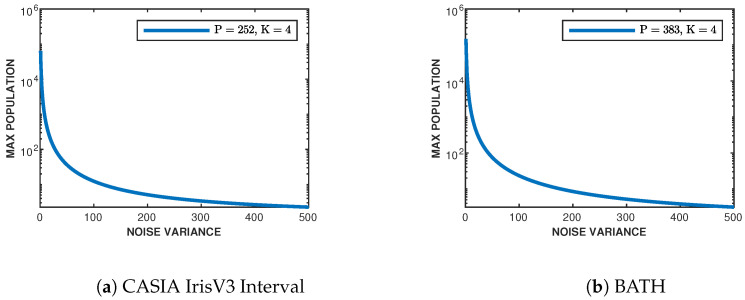
Sphere packing bound for (**a**) CASIA-IrisV3 Interval and (**b**) BATH using the relative entropy metric.

**Figure 7:**
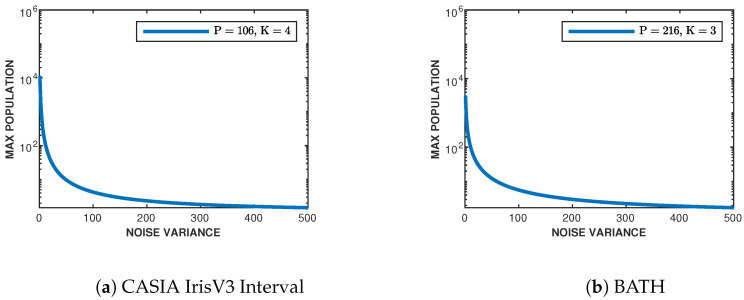
Sphere packing bound for (**a**) CASIA-IrisV3 Interval and (**b**) BATH using the log-likelihood metric.

Now that we have obtained the best-of-fit chi-square distributions for the relative entropy and likelihood imposter histograms, the maximum population can be empirically found through the use of the sphere packing bound presented in Section 2.6. Looking first at likelihoods, the found degrees of freedom and variance are used in (12). For the measure of noise variance, N, this depends on the quality of the iris datasets themselves. Since there is no simple method to find the noise present in the iris classes themselves, the noise variance is varied to reflect possible values (from little to extremely noisy images). Figure 6 shows the resulting bound on the supported maximum population for the CASIA-IrisV3 Interval (Figure 6a) and BATH (Figure 6b) datasets dependent on the given noise variances using relative entropy as a distance metric. The same procedure is implemented on the found log-likelihood fitted chi-square distributions for each dataset, and the resulting bounds are shown in Figure 7. Table 1 shows the maximum population of each dataset given a noise variance.

Given that the noise variance is dependently related to the quality of the iris images contained in the dataset (e.g., motion blur, focus, distance, noise, etc.), the higher the quality of iris acquisition, the smaller the noise variance, and vice versa. This aligns with the results presented in Table 1. We can conclude that as the noise variance increases (image quality decreases), the maximum population we can support in both datasets diminishes. Since the BATH dataset contains higher-quality images than CASIA-IrisV3 Interval, the maximum supported population given the lowest noise variance (N=1) is $1.48\times 10^{5}$ classes for relative entropy and $3.2\times 10^{3}$ classes for log-likelihoods, while CASIA’s is $6.40\times 10^{4}$ classes for relative entropy and $1.15\times 10^{4}$ classes for log-likelihoods. The reason we are seeing a higher supported population for CASIA, given sum-log-likelihoods, is due to the higher degree of freedom in the fitted histogram distribution.

### 3.6. Daugman-like Bound

**Figure 8:**
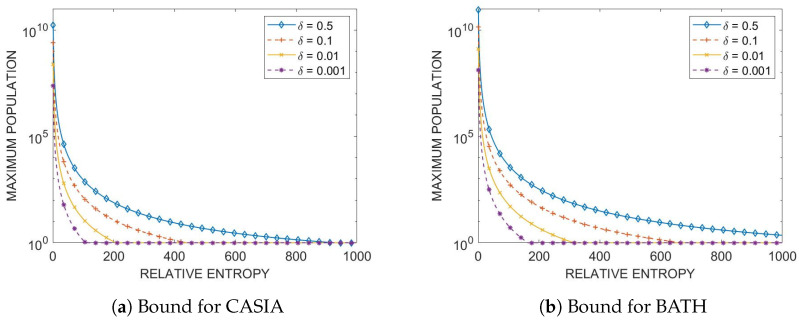
Daugman-like bound for (**a**) CASIA and (**b**) BATH datasets.

Looking once again at the fitted chi-square imposter distributions for relative entropy, we can utilize our Daugman-like bound from (16) to find the maximum population the datasets can achieve at a given image quality of data and a fixed recognition error, δ. To begin, the cumulatives of our fitted distributions, from Section 3.4, $\chi^{2}\left(\tau \right)$ from 0 to τ, are found, where τ is a given relative entropy metric interpreted as a distance between two codewords. Now, using these cumulatives, we can find the FMR from Equation (16) given a particular relative entropy value, τ, and a fixed recognition error, δ (which is varied similar to Daugman [6], to reflect recognition errors of 50%, 10%, 1%, and 0.1%). Figure 8 shows the resulting bounds for the CASIA-IrisV3 Interval and BATH datasets, and is illustrated for fixed values of the relative entropy in Table 2. Note that τ plays the role of the minimum possible distance allowed between two codewords that belong to two different classes. This implies that the distance between two codewords from the same class is approximately τ/2 or less, which is achievable only if enrolled and query data in the form of codewords are of high quality.

Looking at Table 2, we can see that as the relative entropy (the distance between codewords), τ, increases, the maximum population obtainable, given a certain recognition probability, decreases exponentially. Looking ideally at a recognition error of δ=0.001 and τ=1, the maximum population obtainable by the CASIA-IrisV3 Interval dataset is $2.43\times 10^{7}$ and for the BATH dataset, it is $1.30\times 10^{8}$. This result accords with intuition because the BATH dataset is of greater quality than the CASIA-IrisV3 Interval dataset; therefore, it can sustain 20% more classes. To turn an idealized possibility into reality, a low value of relative entropy (minimum distance between two codewords of two different classes or maximum distance between codewords of the same class) can be achieved by ensuring that the quality of all data is as high as possible, which can be accommodated using modern cameras capable of collecting hundreds of frames over a short interval of time followed by the application of a bulk of signal/image processing techniques.

## 4. Conclusions

This work assumes the existence of a mapping from iris data to a space of Gaussian codewords with independent components and presents a basic theoretical methodology to find the maximum population of an iris database in both a closed system perspective, the sphere packing bound, and an enrollment perspective, the Daugman-like bound. Within the presented framework, a measure of the maximum population depends on the quality of the iris images contained in the datasets. For the sphere packing bound, as the noise variance, N, in the iris dataset increases, the maximum population decreases exponentially. The Daugman-like bound presents a similar measure of iris quality on the constraint of the distance measure of relative entropy between two classes’ power spectral densities, which depend on the noise and distortions present in the images. The size of the enrolled population can be increased by choosing a smaller value of the relative entropy (the distance between any two classes), which is achievable when the quality of data is improved. This can be attained due to the more modern data acquisition techniques and data processing applied to the dataset.

As future work, we would like to develop an encoding technique bypassing the vectorization step and directly mapping iris images of different iris classes to Gaussian codewords with iid entries, each with zero mean and variance P, then see how this mapping affects our maximum attainable population. Moreover, we would like to consider a larger dataset, which could allow for us to consider the impact of variations in iris color and patterns and allow for a better statistical validation of the Gaussian-based models.

With the application of the methodology presented above, researchers can better understand the dependence of the capacity of their datasets on the data quality. An appealing approach to achieving this goal is offered by Nguyen et al. [24] whose work presents a constrained design of deep iris networks. This will be the approach that we intend to pursue in our empirical evaluation of iris biometrics capacity. Deep learning approaches proposed in other application fields, such as [25,26], will also be explored as a way of achieving an effective mapping of iris data to a space of Gaussian codewords. 

## Figures and Tables

**Table 1:** Subset of sphere packing bound values given certain noise variance (N) from Figure 6 and Figure 7.

Noise Variance	Relative Entropy		Likelihoods	
	$M_{CASIA}$	$M_{BATH}$	$M_{CASIA}$	$M_{BATH}$
1	$6.40\times 10^{4}$	$1.48\times 10^{5}$	$1.15\times 10^{4}$	$3.2\times 10^{3}$
10	686	$1.54\times 10^{3}$	134	107
50	36	74	9	12
100	12	23	4	5
200	5	8	2	2
300	3	5	2	2
400	2	3	2	2
500	2	3	2	2

**Table 2:** Subset of Daugman-like bound values given a certain relative entropy from Figure 8.

	δ=0.5		δ=0.1		δ=0.01		δ=0.001	
RE	$M_{CASIA}$	$M_{BATH}$	$M_{CASIA}$	$M_{BATH}$	$M_{CASIA}$	$M_{BATH}$	$M_{CASIA}$	$M_{BATH}$
1	$1.68\times 10^{10}$	$8.97\times 10^{10}$	$2.56\times 10^{9}$	$1.36\times 10^{10}$	$2.44\times 10^{8}$	$1.33\times 10^{9}$	$2.43\times 10^{7}$	$1.30\times 10^{8}$
10	$5.73\times 10^{6}$	$3.02\times 10^{7}$	$8.71\times 10^{5}$	$4.50\times 10^{6}$	$8.31\times 10^{4}$	$4.38\times 10^{5}$	$8.27\times 10^{3}$	$4.36\times 10^{4}$
50	$1.21\times 10^{4}$	$6.12\times 10^{4}$	$1.84\times 10^{3}$	$2.30\times 10^{3}$	175	886	17	88
100	902	$4.32\times 10^{3}$	137	657	13	62	2	6
200	77	335	11	50	2	4	2	2
400	8	31	2	4	2	2	2	2
600	2	8	2	2	2	2	2	2
800	2	3	2	2	2	2	2	2
1000	2	2	2	2	2	2	2	2

## Data Availability

The data used for this study are available as described in references [19,20].

## References

[1] Bolle R.M., Pankanti S., Connell J.H., Ratha N.K. (2004). Iris individuality: A partial iris model. Proceedings of the 17th International Conference on Pattern Recognition, ICPR.

[2] Schmid N.A., Nicolo F. (2008). On empirical recognition capacity of biometric systems under global PCA and ICA encoding. IEEE Trans. Inf. Forensics Secur..

[3] Yoon S., Choi S.S., Cha S.H., Lee Y., Tappert C.C. (2005). On the individuality of the iris biometric. Proceedings of the International Conference Image Analysis and Recognition.

[4] Adler A., Youmaran R., Loyka S. (2006). Towards a measure of biometric information. Proceedings of the 2006 Canadian Conference on Electrical and Computer Engineering.

[5] Das P., Plesh R., Talreja V., Schmid N.A., Valenti M., Skufca J., Schuckers S. (2023). Empirical Assessment of End-to-End Iris Recognition System Capacity. IEEE Trans. Biom. Behav. Identity Sci..

[6] Daugman J. (2003). The Importance of Being Random: Statistical Principles of Iris Recognition. Pattern Recognit..

[7] Daugman J. (2004). How Iris Recognition Works. IEEE Trans. Circuits Syst. Video Technol..

[8] Daugman J. (2006). Probing the uniqueness and randomness of iriscodes: Results from 200 billion iris pair comparisons. Proc. IEEE.

[9] Daugman J. (2016). Information Theory and the IrisCode. IEEE Trans. Inf. Forensics Secur..

[10] Daugman J. (2021). Collision Avoidance on National and Global Scales: Understanding and Using Big Biometric Entropy. TechRxiv.

[11] Grother P., Quinn G., Matey J., Ngan M., Salamon W., Fiumara G., Watson C. (2012). IREX III—Performance of Iris Identification Algorithms. https://nvlpubs.nist.gov/nistpubs/ir/2012/NIST.IR.7836.pdf.

[12] Zuo J., Hampel K.M., Schmid N.A. (2021). New Perspectives on Recognition Performance of Daugman’s IrisCode or “Everything is New–it is Well Forgotten Old”. IEEE Access.

[13] Cover T.M., Thomas J.A. (2006). Elements of Information Theory.

[14] Shumway R.H., Stoffer D.S. (2017). Time Series Analysis and Its Applications: With R Examples.

[15] Van Trees H.L. (2001). Detection, Estimation and Modulation Theory: Part 1.

[16] Boole G. (1847). The Mathematical Analysis of Logic, Being a Essay towards a Calculus of Deductive Reasoning.

[17] Kay S.M. (1998). Fundamentals of Statistical Signal Processing. Volume II: Detection Theory.

[18] Van Lint J.H. (1999). Introduction to Coding Theory.

[19] CASIA Iris Image Database. http://biometrics.idealtest.org/.

[20] Monro D.M., Rakshit S., Zhang D. (2007). DCT-Based Iris Recognition. IEEE Trans. Pattern Anal. Mach. Intell..

[21] Zuo J., Schmid N.A. (2010). On a Methodology for Robust Segmentation of Nonideal Iris Images. IEEE Trans. Syst. Man. Cybern. Part B (Cybernetics).

[22] Gonzalez R.C., Woods R.E. (2008). Digital Image Processing.

[23] Burg J.P. (1975). Maximum Wntropy Spectral Analysis. Ph.D. Thesis.

[24] Nguyen K., Fookes C., Sridharan S. (2020). Constrained Design of Deep Iris Networks. IEEE Trans. Image Process..

[25] Obayya M., Arasi M.A., Almalki N.S., Alotaibi S.S., Al Sadig M., Sayed A. (2023). Internet of Things-Assisted Smart Skin Cancer Detection Using Metaheuristics with Deep Learning Model. Cancers.

[26] Dahou A., Aseeri A.O., Mabrouk A., Ibrahim R.A., Al-Betar M.A., Elaziz M.A. (2023). Optimal Skin Cancer Detection Model Using Transfer Learning and Dynamic-Opposite Hunger Games Search. Diagnostics.

